# Item-Specificity and Intention in Episodic Memory

**DOI:** 10.5334/joc.110

**Published:** 2020-09-10

**Authors:** Christina U. Pfeuffer

**Affiliations:** 1 University of Freiburg, Department of Psychology, Freiburg, DE

**Keywords:** episodic memory, binding, cognitive control, item-specificity, intentional action

## Abstract

Schmidt et al.’s (2020) PEP model accurately reflects the complexity of task switching based on bottom-up assumptions and episodic memory, re-evaluating the contribution of commonly presumed top-down processes. Extending it to long-term bindings and their item-specific effects could eludicate puzzling findings regarding the independence of long-term bindings between stimuli, responses, and task-specific categorizations as well as the relation between short-term and long-term bindings. Moreover, ideomotor theories of action control provide a bottom-up basis of incorporating volition and intentional action into the PEP model which is currently restricted to stimulus-based action.

Discussions of top-down and bottom-up contributions to cognitive processes are omnipresent in psychological research (e.g., Abrahamse et al., 2016; Awh et al., 2012; Demanet et al., 2010). Schmidt et al.’s PEP model implements instructions (see Ramamoorthy & Verguts, 2012, for an earlier model) and accounts for multi-goal situations simply by encoding bindings in episodic memory and retrieving them. Based on bottom-up processes, their model captures the complexity of task switching, commonly assumed to be top-down controlled (for reviews see e.g., Kiesel et al., 2010; Koch et al., 2018). The PEP model teases apart bottom-up, episodic contributions to determine “pure” top-down contributions to presumed top-down effects. Modelling short-term bindings, it additionally naturally produced long-term effects like learning curves (e.g., Heathcote et al., 2000; Logan, 1988). In the present commentary, I will speculate about whether Schmidt et al.’s PEP model could also account for item-specific binding^1^ effects and how intentional actions could be incorporated into it.

## Item-specificity

The PEP model stores individual episodic bindings separately, but weights them with a recency factor. This reconciles two theoretical accounts of how long-term stimulus-response bindings are encoded and retrieved: One, theories assuming that singular instances are stored and either instance retrieval or response computation wins the race (e.g., Logan, 1988, 1990), and two, theories proposing that bindings vary in strength depending on the number of pairings between stimulus and response (e.g., Horner & Henson, 2009, 2011; Moutsopoulou et al., 2015; Pfeuffer et al., 2018b). Episodic memory in the PEP represents an integration of the two theories. The PEP model’s ability to accurately reflect long-term binding effects should be further assessed by testing whether it can also produce both item-specific facilitation (mapping repetitions) as well as interference effects (mapping switches; e.g., Horner & Henson, 2011).

Noteably, to model instruction following in a multi-goal situation, Schmidt et al. incorporated current notions that, in addition to responses, category/decision/classification^2^ information becomes bound to stimuli (e.g., Horner & Henson, 2009, 2011; Longman et al., 2018; Moutsopoulou et al., 2015; Pfeuffer et al., 2017, 2018a, b). This perspective comes from research on long-term bindings (multi-trial learning-test lags) showing independent effects of item-specific repetitions/switches for stimulus-response and stimulus-category mappings. Evidence for independent long-term bindings is puzzling given that, in multi-goal situations, correct responses can only be retrieved by categorizing stimuli (e.g., Abrams et al., 2002; Pfeuffer et al., 2017), as reflected in the PEP model. Extending the PEP model, so that stimuli also activate bound episodic memories (episode nodes; see earlier version, Schmidt, 2013b) would reveal whether episodic bindings can naturally account for the observed independence of long-term bindings between stimuli, categories, and responses. This could be a first step in using the PEP model to further assess the relation between short-term and long-term bindings (e.g., Moeller & Frings, 2017a, b).

Another promising extension would be including connections between different exemplars and perceptual formats (e.g., word – picture) of one stimulus (i.e., between input nodes) and between different stimuli of one category (i.e., between decision and input nodes). This way, empirically-observed generalizations of bindings to similar exemplars (e.g., Horner & Henson, 2009), other formats (e.g., Horner & Henson, 2011; Pfeuffer et al., 2018a), and other stimuli of a category (e.g., Longman et al., 2018) could be further examined within the same bottom-up model of episodic memory.

## Intentional Action

At present, although response instructions vary depending on task goals, the PEP model only accounts for stimulus-based actions. Yet, theories of intentional action like ideomotor theories (e.g., Hommel, 2009; Hommel et al., 2001; James, 1890/1981) suggest that (bi-directional) bindings between our actions and their effects, acquired by experiencing their co-occurrence, are the core of intentional action (e.g., Elsner & Hommel, 2001). That is, we anticipate future stimuli (effects) and thereby select appropriate actions to produce them (e.g., Kunde, 2001, 2003; Pfeuffer et al., 2016; Pfister et al., 2013). From the ideomotor perspective that goals, future effects, are essentially equivalent to anticipated stimuli, the PEP model should already be able to account (at least) for some aspects of intentional action (e.g., response-effect compatibility: e.g., Kunde, 2001, 2003). Moreover, previous versions of the PEP model (e.g., Schmidt, 2013a; Schmidt et al., 2016) already included a temporal learning mechanism. It should thus also be assessed whether the PEP model can account for temporal effects observed in intentional action (e.g., Dignath & Janczyk, 2017; Kunde, 2003) with the same bottom-up assumptions regarding episodic memory and temporal learning. Should such simulations be successful, apart from extensions to voluntary task switching discussed by Schmidt et al., their model could also contribute to discussions about the interplay of intentional and stimulus-based, habituous action (e.g., de Wit & Dickson, 2009; Hommel, 2017).

## Expectancy and Certainty

Given the structure of the PEP model, there are two further applications that appear fruitful and could lead to novel insights in the respective areas of research. First, time-based expectancy effects are assumed to rely on learned time-event contingencies (e.g., Aufschnaiter et al., 2018; Thomaschke & Dreisbach, 2015). The PEP model with its incorporated temporal learning mechanism could contribute to an episodic memory-based model of not only temporal, but also time-based expectancy, generate novel assumptions, and ignite further theorizing.

Finally, Schmidt et al. had their goal nodes boost the sensitivity of the corresponding decision nodes. This implementation appears to, as a side effect, reflect decision certainty. Future extensions of the PEP model might consider incorporating subjective judgements (e.g., judgements of certainty relevant to (Bayesian) decision-making, e.g., Daunizeau et al., 2010) as additional responses to determine whether even subjective judgements can partly be accounted for by bottom-up processing and episodic memory.

## Conclusion

As promised, Schmidt et al.’s (2020) model helps erase homunculi and lends itself to extensions that will contribute to re-assessments of presumed top-down processes in other areas of psychological research.

## Footnotes

I will use the term binding as an umbrella term for both short-term and long-term associative links. Whenever the time frame is of relevance, the term will be further specificed by adding short-term or long-term.

The term category will be used in the following for the sake of simplicity.

## References

[1] Abrahamse, E., Braem, S., Notebaert, W., & Verguts, T. (2016). Grounding cognitive control in associative learning. Psychological Bulletin, 142(7), 693–728. DOI: 10.1037/bul0000047 27148628

[2] Abrams, R. L., Klinger, M. R., & Greenwald, A. G. (2002). Subliminal words activate semantic categories (not automated motor responses). Psychonomic Bulletin & Review, 9, 100–106. DOI: 10.3758/BF03196262 12026940

[3] Aufschnaiter, S., Kiesel, A., Dreisbach, G., Wenke, D., & Thomaschke, R. (2018). Time-based expectancy in temporally structured task switching. Journal of Experimental Psychology: Human Perception and Performance, 44(6), 856–870. DOI: 10.1037/xhp0000494 29154625

[4] Awh, E., Belopolsky, A. V., & Theeuwes, J. (2012). Top-down versus bottom-up attentional control: A failed theoretical dichotomy. Trends in Cognitive Sciences, 16(8), 437–443. DOI: 10.1016/j.tics.2012.06.010 22795563 PMC3426354

[5] Daunizeau, J., Den Ouden, H. E., Pessiglione, M., Kiebel, S. J., Stephan, K. E., & Friston, K. J. (2010). Observing the observer (I): meta-bayesian models of learning and decision-making. Plos One, 5(12): e15554 DOI: 10.1371/journal.pone.0015554 21179480 PMC3001878

[6] Demanet, J., Verbruggen, F., Liefooghe, B., & Vandierendonck, A. (2010). Voluntary task switching under load: Contribution of top-down and bottom-up factors in goal-directed behavior. Psychonomic Bulletin & Review, 17(3), 387–393. DOI: 10.3758/PBR.17.3.387 20551363

[7] de Wit, S., & Dickinson, A. (2009). Associative theories of goal-directed behaviour: a case for animal–human translational models. Psychological Research, 73(4), 463–476. DOI: 10.1007/s00426-009-0230-6 19350272 PMC2694930

[8] Dignath, D., & Janczyk, M. (2017). Anticipation of delayed action-effects: Learning when an effect occurs, without knowing what this effect will be. Psychological Research, 81(5), 1072–1083. DOI: 10.1007/s00426-016-0797-7 27638299

[9] Elsner, B., & Hommel, B. (2001). Effect anticipation and action control. Journal of Experimental Psychology: Human Perception and Performance, 27(1), 229–240. DOI: 10.1037/0096-1523.27.1.229 11248937

[10] Heathcote, A., Brown, S., & Mewhort, D. J. K. (2000). The power law repealed: The case for an exponential law of practice. Psychonomic Bulletin & Review, 7(2), 185–207. DOI: 10.3758/BF03212979 10909131

[11] Hommel, B. (2009). Action control according to TEC (theory of event coding). Psychological Research, 73(4), 512–526. DOI: 10.1007/s00426-009-0234-2 19337749 PMC2694931

[12] Hommel, B. (2017). Goal-directed actions In M. R. Waldmann (Ed.), Handbook of causal reasoning (pp. 265–278). Oxford University Press DOI: 10.1093/oxfordhb/9780199399550.013.18

[13] Hommel, B., Müsseler, J., Aschersleben, G., & Prinz, W. (2001). The theory of event coding (TEC): A framework for perception and action planning. Behavioral and Brain Sciences, 24(5), 849–878. DOI: 10.1017/S0140525X01000103 12239891

[14] Horner, A. J., & Henson, R. N. (2009). Bindings between stimuli and multiple response codes dominate long-lag repetition priming in speeded classification tasks. Journal of Experimental Psychology: Learning, Memory, and Cognition, 35(3), 757–779. DOI: 10.1037/a0015262 19379048

[15] Horner, A. J., & Henson, R. N. (2011). Stimulus–response bindings code both abstract and specific representations of stimuli: Evidence from a classification priming design that reverses multiple levels of response representation. Memory & Cognition, 39(8), 1457–1471. DOI: 10.3758/s13421-011-0118-8 21671105 PMC3205272

[16] James, W. (1981). The principles of psychology. Harvard University Press (Original work published 1890). DOI: 10.1037/10538-000

[17] Kiesel, A., Steinhauser, M., Wendt, M., Falkenstein, M., Jost, K., Philipp, A. M., & Koch, I. (2010). Control and interference in task switching—A review. Psychological Bulletin, 136(5), 849–874. DOI: 10.1037/a0019842 20804238

[18] Koch, I., Poljac, E., Müller, H., & Kiesel, A. (2018). Cognitive structure, flexibility, and plasticity in human multitasking—An integrative review of dual-task and task-switching research. Psychological Bulletin, 144(6), 557–583. DOI: 10.1037/bul0000144 29517261

[19] Kunde, W. (2001). Response-effect compatibility in manual choice reaction tasks. Journal of Experimental Psychology: Human Perception and Performance, 27(2), 387–394. DOI: 10.1037/0096-1523.27.2.387 11318054

[20] Kunde, W. (2003). Temporal response-effect compatibility. Psychological Research, 67(3), 153–159. DOI: 10.1007/s00426-002-0114-5 12955506

[21] Logan, G. D. (1988). Toward an instance theory of automatization. Psychological Review, 95(4), 492–527. DOI: 10.1037/0033-295X.95.4.492

[22] Logan, G. D. (1990). Repetition priming and automaticity: Common un-derlying mechanisms? Cognitive Psychology, 22(1), 1–35. DOI: 10.1016/0010-0285(90)90002-L

[23] Longman, C. S., Milton, F., Wills, A. J., & Verbruggen, F. (2018). Transfer of learned category-response associations is modulated by instruction. Acta Psychologica, 184, 144–167. DOI: 10.1016/j.actpsy.2017.04.004 28454893

[24] Moeller, B., & Frings, C. (2017a). Dissociation of binding and learning processes. Attention, Perception, & Psychophysics, 79, 2590–2605. DOI: 10.3758/s13414-017-1393-7 28752283

[25] Moeller, B., & Frings, C. (2017b). Overlearned responses hinder SR binding. Journal of Experimental Psychology: Human Perception and Performance, 43(1), 1–5. DOI: 10.1037/xhp0000341 28004956

[26] Moutsopoulou, K., Yang, Q., Desantis, A., & Waszak, F. (2015). Stimulus-classification and stimulus-action associations: Effects of repetition learning and durability. Quarterly Journal of Experimental Psychology, 68(9), 1744–1757. DOI: 10.1080/17470218.2014.984232 25396708

[27] Pfeuffer, C. U., Hosp, T., Kimmig, E., Moutsopoulou, K., Waszak, F., & Kiesel, A. (2018a). Defining stimulus representation in stimulus–response associations formed on the basis of task execution and verbal codes. Psychological Research, 82(4), 744–758. DOI: 10.1007/s00426-017-0861-y 28391366

[28] Pfeuffer, C. U., Kiesel, A., & Huestegge, L. (2016). A look into the future: Spontaneous anticipatory saccades reflect processes of anticipatory action control. Journal of Experimental Psychology: General, 145(11), 1530–1547. DOI: 10.1037/xge0000224 27797559

[29] Pfeuffer, C. U., Moutsopoulou, K., Pfister, R., Waszak, F., & Kiesel, A. (2017). The power of words: On item-specific stimulus-response associations formed in the absence of action. Journal of Experimental Psychology: Human Perception and Performance, 43(2), 328–347. DOI: 10.1037/xhp0000317 27831720

[30] Pfeuffer, C. U., Moutsopoulou, K., Waszak, F., & Kiesel, A. (2018b). Multiple priming instances increase the impact of practice-based but not verbal code-based stimulus-response associations. Acta Psychologica, 184, 100–109. DOI: 10.1016/j.actpsy.2017.05.001 28511771

[31] Pfister, R., Dignath, D., Hommel, B., & Kunde, W. (2013). It takes two to imitate: Anticipation and imitation in social interaction. Psychological Science, 24(10), 2117–2121. DOI: 10.1177/0956797613489139 23990198

[32] Ramamoorthy, A., & Verguts, T. (2012). Word and deed: A computational model of instruction following. Brain Research, 1439, 54–65. DOI: 10.1016/j.brainres.2011.12.025 22264490

[33] Schmidt, J. R. (2013a). Temporal learning and list-level proportion congruency: Conflict adaptation or learning when to respond? Plos One, 8(11): e82320 DOI: 10.1371/journal.pone.0082320 24312413 PMC3842973

[34] Schmidt, J. R. (2013b). The Parallel Episodic Processing (PEP) model: Dissociating contingency and conflict adaptation in the item-specific proportion congruent paradigm. Acta Psychologica, 142(1), 119–126. DOI: 10.1016/j.actpsy.2012.11.004 23261421

[35] Schmidt, J. R., De Houwer, J., & Rothermund, K. (2016). The Parallel Episodic Processing (PEP) model 2.0: A single computational model of stimulus-response binding, contingency learning, power curves, and mixing costs. Cognitive Psychology, 91, 82–108. DOI: 10.1016/j.cogpsych.2016.10.004 27821256

[36] Schmidt, J. R., Liefooghe, B., & De Houwer, J. (2020). An episodic model of task switching effects: Erasing the homunculus from memory. Journal of Cognition. 3(1): 22, pp. 1–38. DOI: 10.5334/joc.97 PMC7485406 32964181

[37] Thomaschke, R., & Dreisbach, G. (2015). The time-event correlation effect is due to temporal expectancy, not to partial transition costs. Journal of Experimental Psychology: Human Perception and Performance, 41(1), 196–218. DOI: 10.1037/a0038328 25419671

